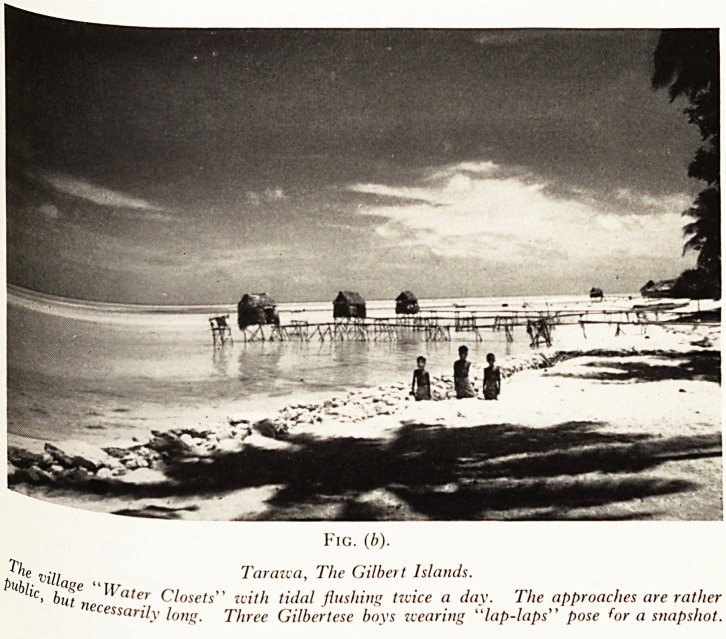# A Tour of the South-Western Pacific

**Published:** 1957-07

**Authors:** A. L. Eyre-Brook

**Affiliations:** Orthopaedic Surgeon, United Bristol Hospitals


### A TOUR OF THE SOUTH-WESTERN PACIFIC

BY

A. L. EYRE-BROOK, M.S., F.R.C.S.

*Orthopaedic Surgeon, United Bristol Hospitals*

I returned from a six-day holiday in the Scilly Isles to be greeted by an invitation from the Colonial Office to visit the South Pacific Islands as a medical visitor. What an opportunity! An immediate acceptance seemed indicated and fortunately the United Bristol Hospitals and the South Western Regional Hospital Board agreed to my being seconded; also my colleagues very kindly accepted the extra work involved. Six weeks later I was away on a ten weeks' trip abroad, as rich in experience as any that I have had the good fortune to enjoy.

*Map of the Pacific Ocean to show the route travelled and the places visited. The tour was centred on the Fiji Islands.*

I was to be a medical visitor and the letter explained the two-fold purpose of my visit; to advise and assist in any medical matter in which I could help, not only in orthopaedic surgery, and to meet as many medical men in these islands as was possible, in order to break down the sense of isolation which easily enfolds those working so far from the centres of population. I had heard of these medical visitors some eight years earlier and had agreed to co-operate when I was asked; the scheme then only embraced Africa. Time passed and we had decided at home that, as far as I was concerned, what I had foreseen had been but a mirage. Now the reality, and the South Sea Islands instead of Darkest Africa. The Director of Medical Services, South-Western Pacific, had requested that the first medical visitor be an orthopaedic surgeon, and in due course the invitation had found its way into my letter-box.

The territory to be visited comprised the Fiji Islands, the Gilbert and Ellice Islands and the British Soloman Islands (see map). New Hebrides was originally included but had to be ruled out as no transport was available, but a short day visit to Tonga was added later.

Travelling was by air throughout the trip, the double-decker Boeing Stratocruiser, the DC7C, the Superconstellation, the Sunderland Flying Boat as the guest of the New Zealand Air Force, the Dakota and the little Rover of the Fiji "Scareways". I had originally been primed for "a rather long journey in a rather small boat" which I later found to be planned to take me from the Gilbert Islands to the British Soloman Islands. Fortunately, the High Commissioner was using the boat and I was returned to Fiji to reach Guadalcanal in the Soloman Islands by travelling to Sydney, Brisbane, Port Moresby and Lae, and spending the last day of the three days' journey hopping from island to island in a Dakota aircraft, passing through Rabaul and reaching Honiara, the capital, just before nightfall.

On retracing my steps to Port Moresby, I stayed there for three days at the request of the Australian Administration, and saw their tuberculous spines and leprosy cases. Medical visitors are comparatively rare on that route and one gets one's specialist advice when opportunity offers. I sometimes represent this trip as a giant "Domiciliary Visit", but it was a long time since the last such visit to some places.

I was very fortunate in being well cared for by many new-found friends, and I usually stayed in their homes and was passed from one to another as I moved about the territory.

Bristol was well represented. Dr. C. Gurd is the Medical Specialist at Suva and the only Medical Specialist in the British South Western Pacific territories; Dr. Downes is the Medical Officer to Nandi Airport; Dr. Bauman in Tonga had been in general practice in Bristol, and two other medical men had worked as house surgeons in Bristol during the last ten years.

The medical service varied from one territory to another, being most advanced in Fiji which, having a European population of over 9,000 and being on the main air route between San Francisco and Australia and New Zealand, is no backwater in this contracting world. Fundamentally, the services are similar in that most people primarily consult a medical man with less training and experience than that provided for in the qualifying medical examinations of most countries. The Assistant Medical Practitioner, trained in Fiji with a course now extending over five years, provides the basic practitioner of medicine in most of the South Pacific Islands. The Central Medical School at Suva, the capital of Fiji, takes students from Samoa, Tonga, the Gilbert and Ellice Islands, the British Solomon Isles and from New Guinea. The school has about 120 students and qualified its first women students last year. In all the Islands, the Assistant Medical Practitioner practises medicine in the villages and in the hospital, and is supervised in both by the Medical Officers, or in the occasional large hospital by the Medical and Surgical Specialists. In some territories where the islands are even more scattered, as for instance in the Solomon Islands and the Gilbert and Ellice Islands, resort has to be had to Dressers—medical aides with far less knowledge and experience, but who comprise the only reasonable unit to be scattered so widely to serve so small a population. In New Guinea, the medical services have been developed along different lines, using Europeans with a modicum of medical knowledge recruited from those who served in the medical services of the three Forces or with previous hospital or first aid experience. These European Medical Assistants have supplied the basic medical services for the scattered population.

The Medical Officer always supervises the services of a wide-area, controlling the work in the large hospitals and the very important Public Health and Preventive Medicine aspects of the whole area. New Guinea is now training many Assistant Medical Practitioners in the Central Medical School at Suva to develop a basic medical service from the many races native to New Guinea.

While in Suva, I took the students on surgical rounds on several occasions and found some quite promising and intelligent students amongst them. At the moment, it is not possible to make any advance from the Assistant Medical Practitioner qualification; it is necessary to start again in a University in New Zealand or Australia. The World Health Organization has taken a great interest in the Central Medical School, Suva, and has provided two extra lecturers in the pre-clinical subjects. There is a strong feeling that progress should be made towards getting the pre-clinical subjects up to a standard acceptable to some university, so allowing a promising Assistant Medical Practitioner later to take a full medical qualification by further study in the clinical subjects alone.

One very promising Assistant Medical Practitioner, practising surgery in one island I visited, was granted an "honorary matriculation" for his services to the Army of his country during the war. I was interested to find that he was thereby enabled to study at Dunedin; the first year he passed his biology, the second his chemistry and the third year he returned to his surgery in his home town. Basic education, and particularly weakness in mathematics, were the reason for the failure of this wholly laudable, if unconventional, academic exercise.

The Central Medical School, Suva, is staffed by some excellent teachers with a great sense of vocation, and it provides a fundamental service to medicine in these islands. The training of Assistant Medical Practitioners is not a temporary expedient, but will continue to provide the basic practitioner in medicine to these myriad of islands, where geography compels the community to live in units too small to provide either the remuneration or the clinical interest sufficient to hold a man or woman more highly qualified.

When I operated in Suva, the anaesthetics were always given by an Assistant Medical Practitioner, and the very pleasant and capable surgeon in Nukualofa, the capital of Tonga, is an Assistant Medical Practitioner. There are many such able practitioners of medicine and surgery among their ranks, but a full medical qualification must demand a basic education which the older generation of Assistant Medical Practitioners has not had the good fortune to enjoy.

The Medical Officer I met was usually quite young and enthusiastic, but practising quite a different brand of medicine than his contemporary at home. Public Health and Preventive Medicine affecting the villages, labour lines, scattered houses and the townships take much of his time. Supervision of the Assistant Medical Practitioners in his area involves much travelling, often on horseback or by boat, and control of his hospital with such surgery as he deems advisable, due allowance being given to the nursing in these smaller units as well as to his own ability, adds further variety. To the European population he often acts as general practitioner, although in many places the primary service is given by the Assistant Medical Practitioner, and geography might well result in his being the sole bastion between his friend and some fell disease which, in England, would be looked upon as almost the exclusive province of some less well-known speciality. I met Medical Officers wholly responsible for almost all the medicine, surgery, obstetrics and all the specialities, not forgetting dental surgery and even an occasional veterinary case: save in the last, his patients include a European section. Evacuation is difficult and erratic and only occasionally enables him to obtain the help of other medical men. The various territories largely deal with their own problems, but the medical and surgical specialists at Suva make it a focal point for consultations or surgery, while other patients go further afield to Australia and New Zealand, from whence are derived many of the white people living in these islands.

The nursing staff comprises European sisters recruited from Australia and New Zealand or the United Kingdom, Fiji-trained nurses from the Suva school of nursing and untrained assistants.

The Sisters of St. Mary provide a wonderful service for the lepers at Makogai in the Fiji group and in the Leper Colony on Guadalcanal. Their cheerful efficient service, so humanely rendered to these unfortunates, has been dramatically rewarded since 1948 by the amazing results which have resulted from the introduction of the sulphone group of drugs.

The disease pattern is very different from our own. Appendicitis and peptic ulcers are exceedingly rare, trauma is not very prominent and, in its severer form in Fiji, is often the result of an attack by an Indian on an Indian; malignant disease is seen in smaller numbers than in Europe, but the population has a lower average age. Undernutrition and anaemia are usually considered to be among the more common ailments suffered by the Indian population in Fiji, more so among the women with their large families. The Fijians are usually much fitter but are very prone to tuberculosis, which is the major problem in these islands. It is difficult to assess the relative incidence of tuberculosis in Fiji between the two races as the Indians, under social and economic stress, will not stay in the chest hospital, while the Fijians, with the security of the village life for all their dependants, occupy the hospital beds, content to accept the treatment and fill the evening air with their delightful music and song. In Fiji, mass radiography and B.C.G. innoculation for children all feature in a vigorous programme designed to control this most serious of their diseases.

In the countries I visited, malaria was absent from Fiji and the Gilbert Islands, but was a very important cause of ill health in New Guinea and the Solomon Islands.

Filariasis is not infrequent, causing lymphangitis and giving positive blood smears, but elephantiasis is quite rare, save among the Ellice Islanders; this late result appears to follow very many years of infection.

Yaws in all the territories is reeling beneath the blow of 1 Mega unit of penicillin to every head of population, prescribed by the World Health Organization and delivered by teams of local Assistant Medical Practitioners under its direction. Is this single blow fatal? We have yet to find the answer to this question, but the lesions are becoming very rare and penicillin has remarkable curative properties in this disease.

Tetanus appears to be much more common than in England, and I asked for an injection of A.T.S. when I came off my Vespa just in case I might prove unlucky. The results of treatment are, however, very good, apart from the invariably fatal neo-natal cases. I came to the conclusion that tetanus was not quite the terrifying disease in Fiji that it still proves to be in England.

A round of the wards produced some unusual cases—the woman with burnt soles of her feet from fire walking, and the man extensively burnt from receiving the steam treatment. He appeared to have been overdone and then hidden away, to become septic, lest the Authorities should prove too severe. The Witch Doctor's curse is still a power in these islands and the ability to survive seems to disappear from healthy men and women when this curse is laid on a Fijian.

I never saw a congenital dislocation of a hip, but club feet are common. Anterior poliomyelitis has touched the Fijian islands lightly but has left more cripples in its wake in the Solomon Islands.

Leprosy was, until recently, strictly segregated in leper colonies, and the lovely island of Makogai in the Fiji group was the largest. The island is divided into the clean and dirty areas: in the former live most of the healthy personnel, skilled and unskilled, who run the colony; the latter is the area to which the lepers are confined. The Sisters and the Padrés live in the "dirty" area, and maintain the wonderful atmosphere of courage and hope that pervades the community. The women lepers of all races live in the women's compound, while the men live in villages—the Indian, the Fijian and the Gilbertese villages; the Samoans and the Ellice Islanders also come to Makogai when afflicted with leprosy. There was only one white woman and she had been in the island for fifteen years; Father Choblet, about whom we read in *A Pattern of Islands*, left two or three years ago after twenty-five years' residence. The men tend their gardens, do woodwork and engage in the rather limited opportunity for paid work for lepers on the island. The women engage in excellent needlework, showing the differing designs and work from the different islands in their decorated counterpanes and pillow-cases—one of the latter with the poignant remark

"Hungry for Love". The Leper Trust Board provides all those extras for which the Army rely on the Red Cross; the occupational therapy workshops for men, the needlework room with the battery of sewing machines for women, the cinema for all.

Treatment has been revolutionized by the sulphone drugs, which have healed all but the sores from chronic secondary osteomyelitis or trophic ulcers Plate VI (*a*). The patients are happy and fit, but are not cured with the ease with which the many lesions are induced to disappear. The criteria of cure until recently were two years with no positive smear—now it has been reduced to one year. The swab from the nose, a drop of blood from the ear and a scraping from any area suspicious of a lesion must be free from lepra bacilli over these long periods. These tests are not over-stringent for a cure, as a case may be consistently negative and yet, on operating on a nodular nerve, it will be found to be teeming with bacilli.

The newer approach to leprosy, better called Hansen's disease to dispel the ora of uncleanliness which surrounds the former title, is to allow the patient to return to his village under sulphone treatment and instructed in elementary precautions, once the lesions are cleared up. In the British Solomon Islands, where leprosy is much more common, this is the practice—it appears to be safe, far more humane and allows the medical department to treat a very much larger number of patients from a scattered community, where the full incidence of the disease is still unknown.

The leper colonies in Makogai and in the British Solomon Islands are run by the Sisters of St. Mary, a Roman Catholic Sisterhood drawn from many countries and doing a magnificent work with such enthusiasm and humanity. In Port Moresby, the London Missionary Society runs the leper colony with similar devotion but they are not so fortunate with the siting of their unit. The work of the Churches in these islands shows the co-operation in the past which has minimized the confusion that results from several denominations evangelizing in one community. In the territories mandated under the United Nations, such restraint tends to be interpreted as infringement of liberty and the result is not helpful to unenlightened peoples receiving the Christian message in so many discordant tones. The Tongans are wholly Methodist, the Fijians are very largely Methodist, while the Anglican Communion concentrates on the British Solomon Islands, the Pro-Cathedral of the Bishop of Melanesia being at Honiara. The far-flung Diocese of the Bishop in Polynesia has its headquarters in Suva, although the number of Anglicans in the Fiji Islands is comparatively few. The Roman Catholic influence is strong throughout the territories, partly by reason of the permanence of the missionaries. Those of the older generation were sent out to the South-Western Pacific for life, and the continuing influence of these men and women has been very great. The world has shrunk since those days and the Roman Catholic missionaries no longer go out to the South-Western Pacific for quite such indefinite periods.

The remainder of the article must be devoted to the people and the scenery, with occasionally a little history to complete the picture.

The people of the Pacific are divisible into three groups. The Polynesians ("inhabitants of many islands") occupy the large triangle between Hawaii, Easter Island and New Zealand; I visited Honolulu and Tonga, whose people are Polynesian and are the most highly developed of the races in the Pacific. The Melanesians ("inhabitants of the dark massive islands") occupy those volcanic islands which stretch from New Guinea, through the Solomons, New Hebrides, New Caledonia to the Fiji Islands. These people are Negroid in type, some very small and exceedingly primitive, as in the Solomons, while the Fijians are a fine race and highly developed; a glance at the map will show you that the Fijians have penetrated further into the Pacific than have any other Melanesians; some of their development also, no doubt, results from the considerable intermarriage with the Tongans. Finally, there are the Micronesians ("inhabitants of small islands") who occupy the many small islands north of the equator, including the Gilberts, which I visited; these people show a Mongoloid tendency to a varying degree. It is interesting to see how the three primary races of the world are represented in the Pacific—the Caucasian, the Negroid and the Mongolian.

Prior to the advent of the missionary to the Pacific Islands there was no written language, and all history was represented by the tales handed down within the tribes. The Polynesians and more advanced Melanesians were organized with chiefs, and even kingdoms, while the more backward Melanesians in the hills of New Guinea still exist in small, closely-knit villages, suspicious of all contact with the outside world and even now hardly ever meeting a white man.

The advent of the white man to the Pacific Islands introduced the idea of sustained and organized work, which was foreign to the custom of these people and to which they reacted, and sometimes continue to react, by what may be described as an attitude of passive resistance. This led the Europeans to import labour, of which the saddest consequence is the large Indian population in Fiji which now outnumbers the native race. It must not be thought that these South Sea Islanders are an indolent people; their accomplishments with Stone Age implements reveal industry and skill; their canoes and some of the fine meeting-houses show a craftsmanship of which they can well be proud, and the Polynesians have some remarkable monuments in stone. Even now, regular work for wages proves unattractive to many who still prefer the village life, which, however, has lost much of its virility in these days of law and order.

These islands boast no indigenous mammals. The wild pigs were probably left by Captain Cook. The fauna is restricted and often the result of importation in fairly recent years.

These South Sea Islanders in former times engaged frequently in wars and indulged in cannibalism. The scarcity of animal protein apart from fish may be a factor in the great prevalence of cannibalism, but there was, undoubtedly, a belief that eating one's vanquished opponent led one to gain his virtues.

### THE FIJI ISLANDS

The Fiji Islands were ceded to Britain in 1874 by Thackombau, a Fiji chief at the time in the ascendancy, who styled himself King of Fiji. The story of the Deed of Cession is interesting as it was motivated partly by the need to pay a debt of £9,000 presented by the American Consul for damage to American property on the Islands. There was also the ever-present danger of further wars with the eastern islands under a powerful Tongan chief called Maafu. After America, Britain and Germany had in turn refused to take over the Fiji Islands, a second approach to Queen Victoria was successful and the Deed of Cession was signed by Thackombau and his fellow chiefs in 1874. These islands number 320, but there are only two really large ones; they are volcanic, the main island, Viti Levu, rising to 4,000 ft. in a central ridge. The prevailing westerly winds drop all their moisture on the windward side, which receives a rainfall of up to 120 in., while the eastern half of the island has a much more agreeable climate with a rainfall of only 70 in. Surrounding the islands are the coral reefs, in places four to five miles from the shore, and elsewhere within half a mile, and it was through these treacherous reefs that Captain Bligh, in his overladen open boat from the *Bounty*, managed miraculously to pass, pursued by two canoes manned by Fijian warriors, from whom they only managed to escape when a heavy downpour of rain reduced visibility to a few yards.

The major portion of the islands consists of bush forest, untamed and unproductive of any reasonable timber, occupying country unsuited for any other purpose than timber growing. The narrow valleys and coastal plains grow vast stretches of cane sugar, producing the most valuable export—worth from £5 millions to £7 millions annually. This industry is worked almost exclusively by the Indians and the cane is bought and crushed by the powerful organization known as the C.S.R., or Cane Sugar Refining Co. Ltd. of Australia.

Gold is mined to the value of about £1,000,000 a year, and copra, or the dried coconut, worth £2,500,000 annually, forms a staple crop, as it does everywhere in the South-Western Pacific.

The people living in Fiji today comprise some 9,000 Europeans, 145,000 Fijians and 165,000 Indians, along with a few thousand Chinese, Euronesians and peoples from other South-Western Pacific islands. Among these latter are the 1,000 Banabans on Rambi Island, and the 80 Ellice Islanders recently transferred to Kioa.

These interesting population movements are the result, in the case of the Banabans, natives of Ocean Island, of the destruction of their island by the quarrying of the phosphatic rock. They acquired sufficient royalties from this industry to purchase Rambi Island in the Fiji group. The Banabans were scattered widely among the Pacific Islands by the Japanese, who occupied Ocean Island between 1942 and 1946, but they were later assembled at Tarawa and moved to their new home in the Fiji group. The Ellice Islanders were moved on account of over-population in their own islands, where the food supply is particularly slender.

The main population is seen to consist of the Fijians, native to Fiji, and the Indians first brought in during the 1880's as indentured labour for the cane sugar industry. As in Natal, they completed their indentures, continued to work hard, brought in their families and multiplied exceedingly until today they outnumber the Fijians and by their earlier marriages, greater fertility and relatively lower infant mortality continue to increase their numerical preponderance. However, their preponderance is not only numerical, their culture is much greater and much older, so they provide many of the professional men in Fiji—the lawyers and the doctors—and possess a good deal of the wealth, although it is very unevenly distributed. They own very little of the land, which soon after the Deed of Cession could no longer be alienated from the Fijians, who, at a disadvantage in so many ways, must yet retain their land vested in the village, tikini or province which form the communal organization of Fijian life. The Indians are well known for their industry, while the Fijian has no long association with work, apart from the communal duties imposed on the villagers by the headman. The Indian has the aggressive striving of the individualist, which leads, perhaps in uncontrolled moments, to some of the more serious trauma in Fiji. It is said that an Indian's worst enemy is another Indian. The Indians live in scattered houses or small family units when engaged in the cane sugar industry, or in the towns where they run much of the business life.

The Fijian is a most attractive character, a cheerful individual of fine physique, given to music and song, friendly in disposition but not given to flights of ambition. When brought up in the Fijian way, he lives in a village, and works in the communal food gardens or on rebuilding the houses as directed by the headman in conjunction with the village council. Crops are grown for consumption and the idea of a cash crop has only recently received attention. Nowadays, a Fijian can pay a tax to exempt himself from communal duties and may acquire land to grow a cash crop, and gain some wealth by his efforts. The Fijian is, however, beset by the dangers of the generous ceremonial of this way of life and much, or all, can be borrowed by relatives, whom it would be quite unseemly to refuse.

Their lives are much enriched by ceremony; the drinking of Yanggona (which the Polynesians call "Kava"), a non-alcoholic beverage obtained from the ground-up root of a pepper tree, plays a big part in Fijian life both on ceremonial occasions of great importance and in the welcoming of any visitor to the village. Distinguished visitors are presented with the Tambua, or whale's tooth, in a ceremony of some duration and detail. The guest may be treated to the spectacle of a Meki or dance, often by men only, and performed seated, the rhythmic swaying of the bodies, oiled for the occasion, and rapid movement of the hands and head, providing a pleasing if unfamiliar spectacle to the European visitor.

The Fijian children are exceedingly friendly and wave vigorously to any passerby, while it is said that when one waves to an Indian child he will look around to see to whom you have been waving.

In appearance the Fijian has thick lips and a rather flattened nose, and yet quite fine and pleasing features. They are very dark skinned with frizzy hair, worn in a mass stretching straight out from the head for 6 in. or more. The hair style was similar for men and women, and until recently this original hair style was essential for a Fijian joining the Police Force. The influence of the Army in the Second World War and more recently in the Malayan Campaign, where the Fijian Battalion played such an outstanding part, has affected the hair style. As many will remember, the Army is somewhat intolerant on this matter and the Fijian hair style had many disadvantages. Many young men have now come to fancy themselves with hair cut short and plastered to the head with a neat parting, and the mop of Fijian hair is becoming less universal, but I was still able to see the Police Force field a Rugby XV, almost every man wearing the traditional head of hair. The Fijian prowess in the Rugby field is well known, the Battalion having an almost unbeaten record in Malaya, where they played the battalions from Great Britain; there is an old-standing rivalry with New Zealand on the Rugby field, and Samoa and other islands take a very active part in a sport that one would not have associated with the South-Western Pacific Islands.

Male dress usually consists of the Sulu—a length of material wrapped round the body like a skirt or kilt. Originally, no doubt, made of the Tapa or bark cloth, it is now of "calico", a term applied to all imported material. Under various names, this unit of dress is universal throughout the South-Western Pacific, although it is naturally gradually being replaced by shorts or something much more elaborate. I was told by a Government Officer in charge of Public Works in the Gilbert Islands that he doubled the work done by his men by putting them in shorts and thus releasing the second hand from attending to the Sulu or "lap lap" as they are called in those islands.

The majority of these pleasant Fijians do not struggle for high attainment but there are a few who have travelled far and attained much distinction in New Zealand or Great Britain, whether in the university or other sphere. These men and women give as much grace and dignity to a gathering as do any of the Europeans, but these Fijians seem as far from the average Fijian villager as we are ourselves, and one wonders how much more able they are to bridge the gap. The women have, of course, been later in travelling and receiving education, so that it has sometimes been difficult for the cultured Fijian to find a wife to share fully in his life. This picture is changing; the Fijian woman is getting a good education and the first woman Assistant Medical Practitioners were qualified last year.

The Fijian, along with all other South-Western Pacific Islanders, had no written language, and the missionaries in first compiling a vocabulary used certain letters which could well be spared to indicate sounds for which we use two letters, an example being "c" to pronounce the sound "th". The sounds "b" and "d" never occur alone, so that "b" and "d" are pronounced "mb" and "nd". The result has been a special value for certain letters applicable only to the Fijian language. This has naturally not been universally accepted and has led to the confusion of two spellings for many place names, e.g. Nandi and Nadi for the airport on Fiji.

### TONGA

Tonga was, unfortunately, only visited for a few hours, but I was able to shake hands with Crown Prince Tungi, the Prime Minister; however, I did not meet the Queen. The Tongans have a much fairer complexion than the Fijians and are typical Polynesians with altogether finer features on European standards. They have a fine physique and, with their famous feasts, can become very large.

Nukualofa, the capital of Tonga, is situated in the southernmost group of islands. I was disappointed to find a small modern city laid out in square blocks, like any new-world town. Many of the older buildings in all these islands were of log frames and coconut leaf thatch, so that the past has largely disappeared in the towns and the New Zealand contractors have built a modern town on the flat island of Tongatabu. But more durable monuments have survived in Tonga and witness to the greatness

FIG. (*a*).

*A Solomon Island woman in the Leper Colony. The ulceration has involved the bones, and such secondarily infected lesions do not heal under Sulphone therapy. Her hands were also involved. The anaesthesia relieves her of pain but contributes to the persistence of the deep ulceration.*

FIG. (*b*).

*Tarawa, The Gilbert Islands.*

*The village "Water Closets" with tidal flushing twice a day. The approaches are rather public, but necessarily long. Three Gilbertese boys wearing "lap-laps" pose for a snapshot.*

of their ancient kings. The royal burial grounds are quadrangular mounds faced with huge blocks of coral rock, but the greatest monument is an arch of coral blocks, the exposed parts of the upright members being 16 ft. high and the cross piece is 19 ft. long, mortised into the tops of the uprights. The visible parts of this single arch must weigh 30–40 tons. Men skilled in the use of rollers and levers, and with a large body of men at their disposal, must have constructed this arch, much as was done at Stonehenge in our own distant past.

The short time I had in Tonga enabled me only to visit the hospital and meet a few people: I certainly left with the feeling that the visit had been altogether too short, but time was limited and when travelling in the South-Western Pacific the return journey must be carefully worked out when planning any visit, and I found that I had to return on the same plane.

### GILBERT ISLANDS

The visit to Tarawa in the Gilbert Islands was for three days and the scenery of these islands, drenched with sunshine, was the South Sea Islands of which I had dreamed. A few degrees north of the equator, it was hot, but the sea breezes always produce a very pleasant climate.

The arrival of the British in 1890, when the islands passed under British rule, interrupted one of the frequent land wars. As can be imagined, the limited land led to much dispute and frequent wars, and it is a regrettable fact that the disputes over land ownership have continued ever since, and since the Second World War yet another Commission has been set up to attempt a solution.

The inhabitants of the islands are Micronesians, small in stature with a slight but definite Mongoloid appearance and dark brown in colour. The Gilbert and Ellice Islands are grouped together and administered together with Ocean Island, but the inhabitants of the Ellice Islands are Polynesians.

There is no running water on a coral island; water is obtained at a few feet under the surface, at places unpleasantly brackish, elsewhere quite pure. It is, of course, filtered from the ocean and lagoon, neither of which can be much more than 200 yards away. The scanty soil on the coral sand or rock supports innumerable coconut trees and a little "taro", a type of root used throughout the Pacific as a staple cereal. Fish form the main source of protein, although nowadays a few pigs and chickens are kept. The only export is copra. The reply I received to the anxiety I expressed on this "single crop" economy was that the Gilbertese can get almost everything from the coconut apart from administrators and doctors, both of which perhaps they might well do without! A sale for the coconut is, therefore, not essential to these islands. The coconut provides the timber and the thatch for houses, the husks and shells for firewood, the coconut meat as food for animal or human consumption, and the contents of the immature nut is excellent for the infant; the spathe can be cut to provide a drink, which, when fermented, gives a very potent draught. What more does one need but the fish on these pleasant islands?

The Native Magistrate was holding Court in the small islet where I stayed and it is the custom for all to dismount when passing the courthouse. The bicycle is a very satisfactory means of transport in an island where the highest point is some sixteen feet above sea level.

The smaller gaols for lesser offences run very economically by a system allowing the prisoners home each week-end; they return with enough supplies to keep them through the coming week.

The scenery is delightful, the lagoons having so many shades of blue and green, the coconut groves and the pure white coral sand completing the setting. Apart from sunsets, there are few reds in the picture. Plate VI (*b*).

I stayed with the Medical Officer, until lately a well-established partner in a practice in Cornwall, but he was only in his early forties and the distant lands seem to have beckoned to his adventurous spirit and here he was, transported to be Senior Medical

Officer in Tarawa, with the late Sir Arthur Grimble's cook (male) in charge of his menage. His wife is soon to join him under the Southern Cross.

The Senior Medical Officer has usually one other Medical Officer with whom to cover the needs of the 38,000 inhabitants of the Gilbert and Ellice Islands, occupying over thirty island units. The difficulties of such a command are better understood when one realizes that each island is an atoll, comprising a string of little islands, never more than 400 yards wide, surrounding a central lagoon which may be ten to fifteen miles across. Transport is difficult to arrange and the only regular service is the recruitment ship from the phosphate industry on Ocean Island, which annually recruits fresh labour while returning those who have worked for the previous twelve months. Medical supervision to such small island units extends the services of the Assistant Medical Practitioner and the Dresser to their full, one Medical Officer being only able to make infrequent visits, while the second has to control the hospital at Tarawa. These medical officers working far from any regular sea or air route were encouraged by the building of a new hospital, now nearing completion: the purchase of equipment and a new X-ray unit was one of the matters engaging their attention. The hospital provides for the minor and major ailments, along with the tuberculous chests or bone disease—as always a major problem in these territories. There was the mental hospital for the uncontrollable case alongside, and the leper community a little way off. There was, fortunately, an English matron and one sister in this hospital, thus assuring the Medical Officer of a standard of nursing which can never be relied upon as yet in the South Pacific without careful European supervision. A "European" dispenser gave valiant support, to complete the hospital team.

### BRITISH SOLOMON ISLANDS

From the Gilbert Islands I returned to Fiji, and so via Sydney and New Guinea to Honiara in the Solomons. This trip, hopping from island to island, showed me the innumerable races in this part of the world—short and tall, brown and coal-black. They were, undoubtedly, the most primitive people that I had seen, and there were stories of inhabitants of villages high in the mountains who had hardly ever seen the white man. The population of the British Solomon Islands numbers about 80,000, of which about 38,000 live in one large island, Malaita. Some of the islands by reason of their altitude have less land for native villages, but that does not appear to be the primary reason for the congestion in this island of Malaita. Since the war there has been considerable resistance to the European rule in this island, as also in New Guinea, but more co-operation has been forthcoming in the last few years. The economy relies entirely on the coconut, although geologists are active in the search for mineral wealth in these volcanic islands. The names in this part of the world recall the Japanese war—Guadalcanal, Rabaul, Lae, the Owen Stanley Mountains reaching 23,000 ft., and Port Moresby—and there are very frequent reminders of this recent war in the wrecks that are scattered around the coasts, the rusting machines of war, the many aerodromes and some large cemeteries. For many years after the war, scrap iron was the most valuable export from the British Solomon Islands. Rehabilitation of the coconut plantations is now far advanced and the coconut again dominates the scene.

Honiara is the capital of the British Solomon Islands and has a rapidly growing community. Here live most of the Government Officers and their families, but the European community remains small. There are, for instance, only four doctors in the service, two living in Honiara, one largely controlling the hospital while the senior is the Senior Medical Officer with the administrative and public health work as his main preoccupation. There is a fair sized Chinese community in Honiara, running many of the shops and other small businesses. Honiara was selected as the capital after the War, the American buildings, aerodromes and roads providing sufficient inducement. The absence of any deep-water port in the vicinity, with the consequent reliance on lighterage for unloading every ocean-going vessel is, however, proving a serious drawback to the siting of the administrative centre and only town on the islands.

After a week in Guadalcanal, I retraced my steps as far as Port Moresby, the chief town and administrative centre of Papua—New Guinea. Here, 6,000 Australians and New Zealanders enjoy complete absence of Income Taxation, an inducement sufficient to encourage many to accept the less pleasant climate and more pioneering conditions in these Territories, administered by Australia under a United Nations Trust. At the invitation of the Medical Department, I stayed there three days and saw the tuberculous spines and the leprosy cases which presented orthopaedic problems.

The newspapers at that time carried news of raiding by some primitive mountain races with strong suspicion of cannibalism. It appeared that it would take some two weeks to penetrate into the area in order to attempt to obtain the facts of the situation. The country is mountainous and hostile, the people are very primitive and often warlike, and civilizing influences are only just touching them. Australia struggles with the many problems involved and is somewhat unsympathetic to suggestions from the United Nations that a date should be given when Papua–New Guinea will become a sovereign state.

Nine weeks in the South-Western Pacific were drawing to a close and I left for home, very grateful for the opportunity of seeing so much and hopeful that one day I might be able to return to these beautiful islands and colourful peoples.

## Figures and Tables

**Figure f1:**
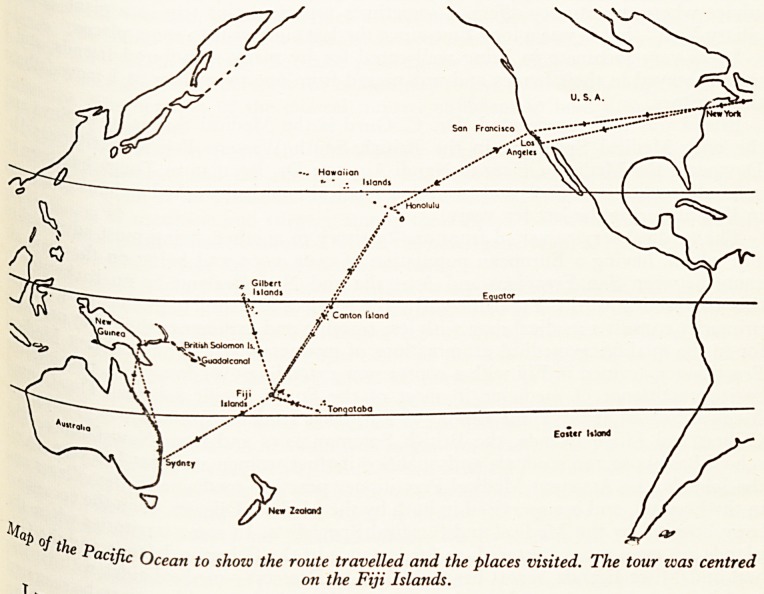


**Fig. (a). f2:**
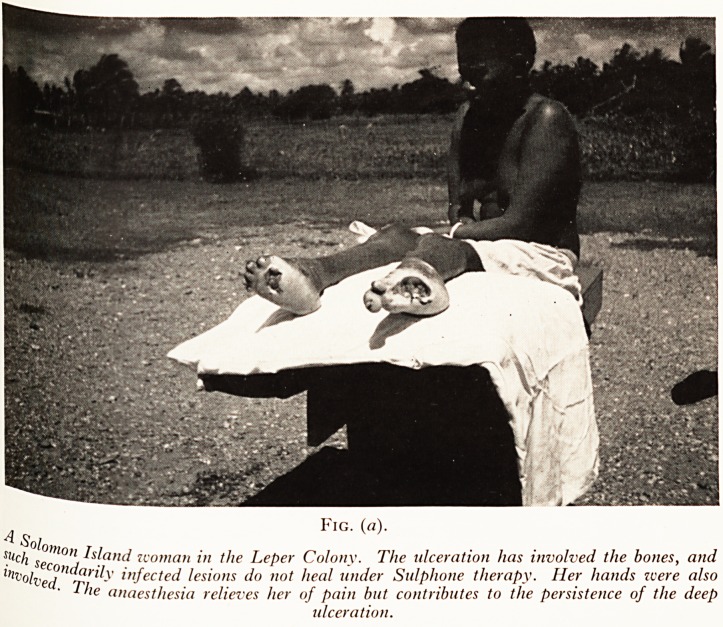


**Fig. (b). f3:**